# The Preparedness of Hospital Emergency Departments for Responding to Disasters in Iran; a Systematic Review and Meta-Analysis

**Published:** 2019-10-08

**Authors:** Mosayeb Kazemzadeh, Elham Shafiei, Katayoun Jahangiri, Kosar Yousefi, Ali Sahebi

**Affiliations:** 1 MSC in Health CareManagement, Ilam University ofMedical Sciences, Ilam, Iran.; 2 Clinical Research Development Unit, Shahid Mostafa Khomeini hospital, Ilam University of Medical Sciences, Ilam, Iran.; 3 Department of Health in Disasters and Emergencies, School of Public Health and Safety, Shahid Beheshti University of Medical Sciences, Tehran, Iran.

**Keywords:** Disasters, disaster medicine, emergency service, hospital, meta-analysis as topic

## Abstract

**Introduction:**

Hospitals are the most important infrastructures of any society. The hospital emergency department is one of the most important wards of hospitals in response to disasters. The purpose of this study was to evaluate the preparedness of hospital emergency departments in response to disasters in Iran via a systematic review and meta-analysis.

**Methods:**

This study was a systematic review and meta-analysis. The literature search was conducted in the national and international databases including SID, Magiran, Irandoc, Google scholar, Medline, Scopus, and ISI. Valid Persian and English keywords were used to extract articles related to the preparedness of hospital emergency departments in response to disasters. The STROBE checklist was used to evaluate the quality of the articles, and the I_2_ index was used to assess heterogeneity among the studies. Statistical analyses were conducted using STATA14 software.

**Results:**

In this study, 185 articles were initially recruited. Meta-analysis was finally performed on 4 articles selected based on inclusion criteria. The analysis included a total of 51 hospitals in Iran. According to our results, the mean preparedness of hospital emergency departments in response to disasters was calculated as 54.64% (95% CI = 41.15-68.13, I^2^ = 0.0%; p = 0.727).

**Conclusion:**

The results of this study showed that the average level of preparedness of hospital emergency departments in Iran to respond to disasters was moderate to high. Therefore, planning and actions should be considered based on the guidelines and accreditation standards to enhance the preparedness of hospital emergency departments in response to disasters.

## Introduction

Hospitals are among the most important infrastructures of any society. The constant activity of hospitals is essential for providing health services to the injured, both in normal conditions and in disasters (1). In addition to inflicting individuals, disasters have many impacts on the functional, structural and non-structural components of hospitals (2). Hospital preparedness is a part of Disaster management cycle. Disaster management preparedness consists of 9 components, the most important of which is planning. Planning includes policies and programs to minimize disaster consequences. Hospitals need to remain fully operational during disasters, and the assessment of hospital readiness is essential to ensure this issue (3). Studies have reported moderate levels of structural, non-structural and functional safety in most Iranian hospitals to respond to disasters. Therefore, there is a need for proper planning and actions to improve the hospital safety level (4). A study conducted on 224 hospitals in Iran in 2014 showed that the majority (54.5%) of Iran's hospitals had high vulnerability to disasters (5). Among hospital wards, the emergency department is a key section in response to disasters. This is because emergency departments play a vital role in providing 24-hour acute care services to outpatients, inpatients, and those in immediate need for assistance (6). The personnel of emergency departments are hospital’s first responders to disasters and therefore, need to be appropriately prepared (7). The emergency department is the gateway for patients to enter the hospital. On the other hand, hospitals face a wide range of disasters and are responsible for responding to a variety of emergencies ranging from traffic accidents to terrorist attacks. Hence, the preparedness of emergency department should not be neglected facing such incidents (8). At the time of disasters, the efficiency of emergency departments to manage Mass casualty incidents (MCI) depends on the availability and adequacy of human and financial resources (9). The purpose of this study was to evaluate the preparedness of hospital emergency departments in Iran for responding to disasters via a systematic review and meta-analysis.

## Methods

The present study was conducted based on the guidelines of Preferred Reporting Items for Systematic Reviews and Meta-Analyses (PRISMA) (10). To prevent bias, search, selection of studies, quality evaluation and data extraction were conducted by two researchers, independently in each stage. An agreement on the obtained data was finally achieved through group discussions. 

***Search strategy***

The electronic search was conducted in Irandoc, SID, Magiran, Scopus, PubMed (MEDLINE), Embase, and Web of science databases and manual search was done in Google Scholar using valid English keywords and their Persian equivalents. The keywords included emergency ward, emergency unit, emergency room, hospital, response, emergency preparedness, emergency readiness, disaster, hospital emergency preparedness, and Iran. The keywords were combined using (AND) and (OR) operators. The search strategy in PubMed used the following terms: ((((("civil defense"[MeSH Terms] OR ("civil"[All Fields] AND "defense"[All Fields]) OR "civil defense"[All Fields] OR ("emergency"[All Fields] AND "preparedness"[All Fields]) OR "emergency preparedness"[All Fields]) OR (("emergencies"[MeSH Terms] OR "emergencies"[All Fields] OR "emergency"[All Fields]) AND readiness[All Fields])) AND response[All Fields]) AND ("disasters"[MeSH Terms] OR "disasters"[All Fields] OR "disaster"[All Fields])) AND ("hospitals"[MeSH Terms] OR "hospitals"[All Fields] OR "hospital"[All Fields]) AND ("iran"[MeSH Terms] OR "iran"[All Fields])) . The timespan of gathering studies was limited to the end of July 2019.

***Inclusion criteria***

The inclusion criterion was descriptive cross-sectional studies that reported the rate of preparedness of emergency departments of Iranian hospitals in response to disasters based on the checklist presented by the world health organization (WHO) (11). The WHO hospital emergency response checklist consists of 9 sections and 90 questions. The average overall preparedness score ranged from 0 to 100. The mean scores of 0-20, 21- 40, 41-60, 61-80 and 81-100 indicated very poor, poor, moderate, good and excellent readiness, respectively (12). Studies in both English and Persian were included.

***Exclusion criteria***

Studies evaluating the preparedness or safety of hospitals in response to disasters as well as those evaluating the preparedness of emergency departments using tools other than the WHO instrument were excluded.

***Qualitative assessment***

The quality assessment of the studies was performed using the standard 22-item STROBE checklist (13). The minimum and maximum obtainable scores using this checklist were 0 and 44, respectively. Studies that acquired a minimum score of 16 were selected for meta-analysis.

***Data Extraction***

Initially, articles with unrelated titles were removed and then the abstracts and full texts of related articles were reviewed according to the inclusion criteria. The required data was extracted from the included articles using a pre-prepared checklist. The collected data included the first author, the location of study, the year of study publication, type of the study, number of evaluated hospitals, instruments used in the study, and finally the mean level of emergency department preparedness in response to disasters.

**Figure 1:**
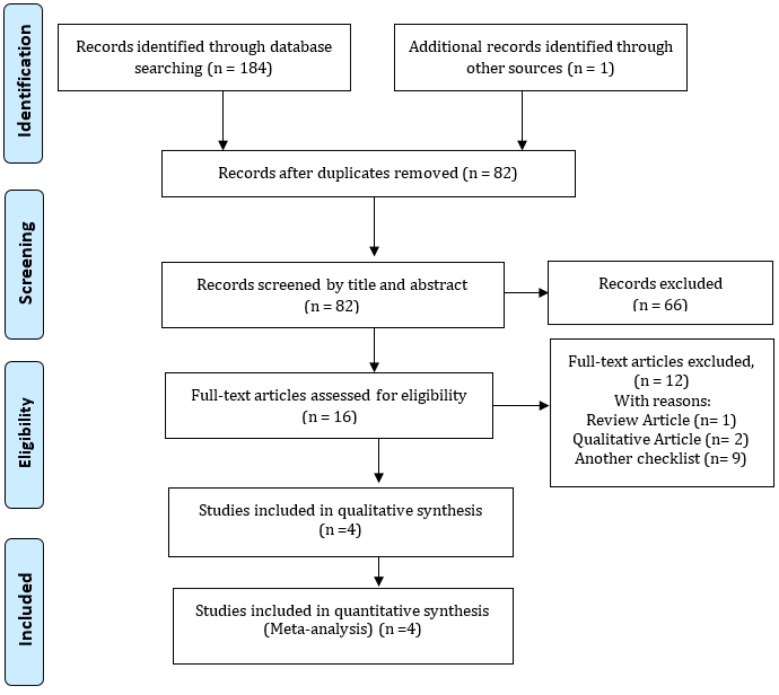
PRISMA flow diagram of the study

**Table1:** The characteristics of the selected studies in meta-analysis

Mean %	Quality	Instrument	Hospitals*	Study type	Year	Place	First author
70.72	Good	WHO	6	CS	2014	Ghazvin	Yousefli (12)
44.17	Good	WHO	13	CS	2015	Karaj	Hasanpoor(20)
54.26	Good	WHO	18	CS	2018	Tabriz	Janati (21)
56.93	Good	WHO	14	CS	2018	Tehran	Seyedin (22)

*: Number of hospitals. CS: cross-sectional, WHO: world health organization checklist.

**Figure 2:**
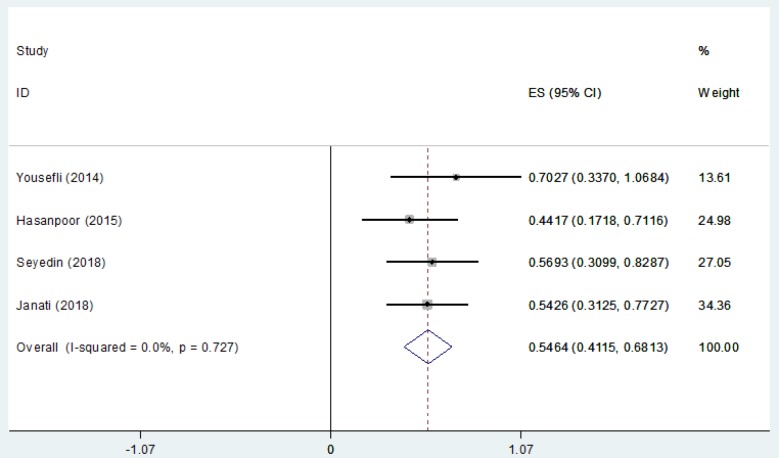
The forest plot of the overall and individual percentages of emergency department preparedness of Iranian hospitals for responding to disasters with 95% confidence interval

***Statistical analysis***

Since the average level of preparedness of hospital emergency department in response to disasters was extracted based on the number of evaluated hospitals, binomial distribution was used to calculate the variance of each study. The weighted average was used to combine the preparedness levels in different studies. Each study was weighted inversely corresponding to its variance. The heterogeneity index of studies was calculated as 0.0%, which was in the range of low heterogeneity indices (i.e. I^2^ indices <25%, 25 to 75%, and >75% indicate low, medium and high heterogeneities, respectively) (14). Therefore, the fixed effects model was used for meta-analysis. In the present study, Egger’s test was conducted to evaluate the possibility of publication bias. The data were analyzed using STATA software (version 14).

## Results

In the initial search, 185 related studies were identified. Of these, 181 studies were excluded due to not fulfilling the inclusion criteria. Finally, 4 cross-sectional studies evaluating 51 hospitals between 2015 and 2018 entered quality assessment and meta-analysis. Figure 1 shows the process of study selection. Finally, 4 studies with good quality entered the meta-analysis. All the included studies used the Hospital Emergency Response checklist presented by WHO. Table 1 displays the general specifications and the data extracted from each study. According to our results, the overall preparedness of emergency departments of Iranian hospitals in response to disasters was 54.64% (95% CI = 41.15-68.13, I^2^ = 0.0%, p = 0.727) indicating a moderate to high level. The highest and lowest levels of preparedness were reported in the studies of Yousefli *et al.* in Qazvin and Hasanpoor *et al.* in Karaj, respectively. The Result of Egger’s test showed that the effect of publication bias was not significant (p = 0.396). 

## Discussion

In the present review, cross-sectional studies reporting the preparedness of emergency departments in response to disasters in 51 Iranian hospitals were analyzed. These studies used the standard checklist developed by the WHO. Overall, the preparedness of hospital emergency departments in response to disasters was 54.64% (95% CI = 41.15-68.13, I^2^ = 0.0%, p = 0.727) indicating a moderate to high level of preparedness. In a study in 2016, Asefzadeh et al. (4) showed that overall preparedness for facing disasters was at moderate level in hospitals in Iran. The overall safety level of Ilam health centers in 2016 was estimated as 22.79%(15). The results of a study by Jalali et al. comparing hospital disaster preparedness between Iran and Sweden showed that Swedish hospitals were at level “A”, while Iranian hospitals attained level “B”. There was no relationship between hospital readiness level and neither the hospital’s size or affiliation. However, the level of hospital readiness was significantly related to the social and economic levels of each country. Furthermore, poor hospital readiness was due to the lack of contingency plans and the inadequacy of resources (16). Considering the vital role of hospitals in treating the injured and reducing mortality in disasters, hospitals should be prepared to respond to disasters regardless of the economic status of each country. The results of another study by Amiri et al. (17) showed that the hospital preparedness in response to disasters was at moderate level in 53 hospitals in northern Iran. In the recent report, it was highlighted that educational programs, hospital resilience, drills, and exercises significantly contributed to improving hospital readiness. In a study, Aladhrai et al. examined the readiness of Yemen hospitals between 2011 and 2013 using the WHO hospital emergency response checklist and showed that no significant improvement was made in Yemen hospitals preparedness against disasters during this period. In fact, the recent report revealed poor performance of all elements that were important for hospital preparedness such as management, surge capacity, and safety. This fact highlights the importance of implementing strategic plans, guidelines, and procedures by health authorities to promote disaster preparedness (18). The results of a study in 2016 by Ingrassia et al. who evaluated the response to disaster of emergency departments at 15 hospitals in Italy showed that 12 hospitals were inadequately prepared. Based on the WHO hospital emergency response checklist, the average preparedness level was lower than normal in the evaluated hospitals (19). Factors affecting the preparedness level of emergency departments included staff, equipment, and systems (8). Following disasters, the emergency departments of hospitals suddenly become overcrowded due to mass casualties, so they must be prepared to respond to the situation. The results of several studies showed that the preparedness of hospitals to respond to disasters in Iran was at moderate level. Therefore, given the high incidence of disasters in Iran, health policymakers should plan appropriate strategies and take adequate measures to improve disaster preparedness at all management levels.

## Limitations

There were very few studies that reported the status of hospital emergency response to disasters using the WHO checklist.

## Conclusion:

According to the findings of the present review, the overall preparedness level of emergency departments of Iranian hospitals in response to disasters was moderate to high. In addition, the level of hospital preparedness in response to disasters was also moderate in Iran. Therefore, given the high incidence of disasters in Iran, health policymakers should take appropriate actions and implement required plans at all management levels using guidelines and accreditation standards to enhance the preparedness of hospital emergency departments in response to disasters.
